# Draft Genome Sequence of *Bacillus cereus* LA2007, a Human-Pathogenic Isolate Harboring Anthrax-Like Plasmids

**DOI:** 10.1128/genomeA.00181-17

**Published:** 2017-04-20

**Authors:** Angela Pena-Gonzalez, Chung K. Marston, Luis M. Rodriguez-R, Cari B. Kolton, Julia Garcia-Diaz, Amanda Theppote, Michael Frace, Konstantinos T. Konstantinidis, Alex R. Hoffmaster

**Affiliations:** a School of the Biological Sciences, Georgia Institute of Technology, Atlanta, Georgia, USA; b Bacterial Special Pathogens Branch, Division of High-Consequence Pathogens and Pathology, National Center for Emerging and Zoonotic Infectious Diseases, Centers for Disease Control and Prevention, Atlanta, Georgia, USA; c School of Civil & Environmental Engineering, Georgia Institute of Technology, Atlanta, Georgia, USA; d Department of Infectious Diseases, Ochsner Clinic Foundation, New Orleans, Louisiana, USA; e Department of Internal Medicine, University of Queensland, Brisbane, Queensland, Australia; f Biotechnology Core Facility Branch, Division of Scientific Resources, National Center for Emerging and Zoonotic Infectious Diseases, Centers for Disease Control and Prevention, Atlanta, Georgia, USA

## Abstract

We present the genome sequence of *Bacillus cereus* LA2007, a strain isolated in 2007 from a fatal pneumonia case in Louisiana. Sequence-based genome analysis revealed that LA2007 carries a plasmid highly similar to *Bacillus anthracis* pXO1, including the genes responsible for the production and regulation of anthrax toxin.

## GENOME ANNOUNCEMENT

*Bacillus cereus* strains containing genetic determinants that confer pathogenic capabilities similar to those found on *Bacillus anthracis* have been recently described (1–5). In *B. anthracis*, virulence determinants are carried on two large plasmids, pXO1 and pXO2, including the genes necessary to produce the anthrax toxin (*lef*, *cya*, and *pagA*) and a d-polyglutamic acid capsule (*capBCADE*) allowing the pathogen to evade host immune response. Plasmids similar to pXO1 and pXO2 have been found in a number of previously sequenced *B. cereus sensu lato* strains causing anthrax-like diseases (1–5). It has not been determined whether or not lateral transfer has occurred between members of *B. cereus* and *B. anthracis*, the direction of such a transfer, or the phylogenetic relationships of the strains based on the plasmid sequences. To gain deeper insights into the evolution of *B. cereus* strains causing anthrax-like diseases, we present the draft genome sequence of LA2007, a bacterial pathogen isolated from a fatal pneumonia case in a female welder from Louisiana.

DNA was extracted from overnight culture on Trypticase soy agar with 5% sheep blood using the Maxwell 16 Promega instrument, and sequencing was performed on an Illumina GAIIx platform. Read quality control, assembly, and annotation were performed as described by Tsementzi et al. (6).

The assembled genome of LA2007 consisted of 67 contigs, with a G+C content of 35% and a total genome size of 5,224,740 bp. The estimated percent completeness and contamination were 99.15% and 0.28%, respectively. The genome was predicted to contain a total of 5,777 putative protein-coding sequences, 15 rRNA operons, and 57 tRNA genes. The calculated average nucleotide identity (ANI) (7) of LA2007 was 94.76% against *B. anthracis* Ames while it was 99.99%; against similar *B. cereus* strains, i.e., strains G9241 (2), 03BB87 (3), and BcFL2013 (1), previously associated with severe pneumonia and cutaneous infections.

Characterization of plasmid gene content showed that the genome of *B. cereus* LA2007 contains a pXO1-like plasmid assembled in eight contigs, each one of them showing at least 99.70% ANI and 80% gene coverage with *B. anthracis* Ames pXO1. The results with *B. anthracis* Ames pXO1 suggest that LA2007 (or its ancestors) might have horizontally acquired the pXO1 plasmid from *B. anthracis* (since the ANI of the plasmid is higher than that of the main chromosome), although detailed phylogenetic analysis will be required to robustly test this finding. A comparison with plasmid pBCXO1 from strains G9241, 03BB87, and BcFL2013 revealed an ANI of ≥99.99% in all three cases, similar to the chromosomes mentioned above. A plasmid homologous to pBc210 reported in *B. cereus* G9241 was also identified in LA2007. The plasmid in LA2007 was assembled in nine contigs and showed 99.98% ANI compared to the plasmid of G9241. Anthrax toxin genes and complete genes for the production of hyaluronic acid synthases (*hasACB*) and exopolysaccharides (*bpsHGFEDCBAX*) were also identified in LA2007 suggesting its ability to produce protective capsules.

### Accession number(s).

This whole-genome shotgun project has been deposited at GenBank under the accession no. MUBB00000000. The version described in this paper is version MUBB01000000 (BioProject ID PRJNA368734).
